# Orthobiologic Products: Preservation Options for Orthopedic Research and Clinical Applications

**DOI:** 10.3390/jcm13216577

**Published:** 2024-11-01

**Authors:** William H. Fang, C. Thomas Vangsness

**Affiliations:** 1 Department of Orthopedic Surgery, Valley Health Systems, 620 Shadow Lane, Las Vegas, NV 89106, USA; 2 Department of Orthopedic Surgery, Keck School of Medicine of USC, Los Angeles, CA 90033, USA

**Keywords:** biologics, stem cells, cryopreservation, lyophilization, orthopedic bioproducts

## Abstract

The biological products used in orthopedics include musculoskeletal allografts—such as bones, tendons, ligaments, and cartilage—as well as biological therapies. Musculoskeletal allografts support the body’s healing process by utilizing preserved and sterilized donor tissue. These allografts are becoming increasingly common in surgical practice, allowing patients to avoid more invasive procedures and the risks associated with donor site morbidity. Bone grafting is one of the most frequently used procedures in orthopedics and traumatology. Biologic approaches aim to improve clinical outcomes by enhancing the body’s natural healing capacity and reducing inflammation. They serve as an alternative to surgical interventions. While preliminary results from animal studies and small-scale clinical trials have been promising, the field of biologics still lacks robust clinical evidence supporting their efficacy. Biological therapies include PRP (platelet-rich plasma), mesenchymal stem cells (MSCs)/stromal cells/progenitor cells, bone marrow stem/stromal cells (BMSCs), adipose stem/stromal cells/progenitor cells (ASCs), cord blood (CB), and extracellular vesicles (EVs), including exosomes. The proper preservation and storage of these cellular therapies are essential for future use. Preservation techniques include cryopreservation, vitrification, lyophilization, and the use of cryoprotective agents (CPAs). The most commonly used CPA is DMSO (dimethyl sulfoxide). The highest success rates and post-thaw viability have been achieved by preserving PRP with a rate-controlled freezer using 6% DMSO and storing other cellular treatments using a rate-controlled freezer with 5% or 10% DMSO as the CPA. Extracellular vesicles (EVs) have shown the best results when lyophilized with 50 mM or 4% trehalose to prevent aggregation and stored at room temperature.

## 1. Introduction

The biological products used in orthopedics include musculoskeletal allografts, such as bones, tendons, ligaments, and cartilage, as well as biological therapies, including platelet-rich plasma (PRP), bone aspiration, micronized adipose tissue, placental products, and other expanded cell-derived therapies [1]. Musculoskeletal allografts serve to augment the body’s healing process through the use of preserved and sterilized healthy donor tissue. These allografts are becoming more commonplace in the operating room, and their use allows patients to avoid a larger surgical procedure and the potential for damaging another part of the body harvesting autograft tissue. Bone grafting is one of the most frequently used procedures not only in orthopedics but in traumatology, oral, and maxillofacial surgery [2,3]. It is estimated the global expenditure in bone grafting and orthobiologics is estimated at USD 5.5 billion [4].

Biologic approaches seek to improve clinical outcomes by enhancing the body’s natural capacity to heal and/or fight areas of inflammation. They serve as an alternative to a surgical operation [5]. There have been promising preliminary results from animal trials [6] and small-scale clinical trials [7]; however, the field of biologics still lacks robust clinical evidence supporting their efficacy. Their exact mechanism of action is still under investigation, and many studies have pointed out anti-inflammatory or immunomodulatory properties [8]. These treatments, which include stem cells and related cellular therapy products, have a large market value of USD 10.2 billion in 2021 that is forecasted to reach USD 20.87 billion in 2025 [9].

**Figure 1:**
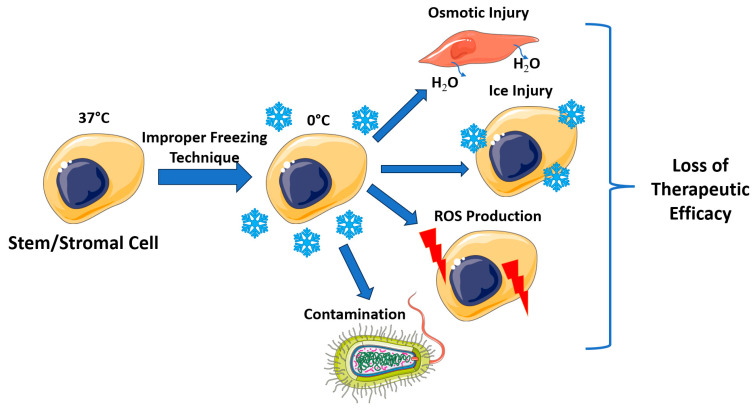
Consequences of improper cell storage.

It is crucial that treating physicians comprehend tissue and cellular preservation and storage techniques to enhance the accessibility of biological therapies. Preserved and stored cells can undergo further analysis for histocompatibility, enabling the development of genetically modified clones. Additionally, preservation and storage options create the potential for cell banks that can store biological products for future use, facilitating inventory control and mitigating aging issues with stem/stromal cells [10]. Extending the shelf life of cell therapies enables additional safety and quality control testing before utilization. Conversely, improper cell storage may lead to a loss of viability, contamination risks, and diminished therapeutic efficacy (Figure 1).

A comprehensive literature search was conducted in September 2023 on PubMed, MEDLINE, and the Cochrane Library, using terms such as “biologics AND cryopreservation technique”, “biologics AND vitrification”, and “biologics AND lyophilization”. Subsequent searches on specific types of biologics included keywords like “PRP, platelet-rich plasma, and plasma”, “MSC, mesenchymal stem/stromal cells, progenitor cells, BMSC, bone marrow stem cells, ADSC, and adipose-derived stem cells”, “Cord products, gestational tissue, placental tissue, and cord blood”, and finally, “exosomes and extracellular vesicles”, replacing the term “biologics”. The objective of this review article is to assess and critique the published storage and preservation techniques for cellular therapies. The preservation techniques discussed in this manuscript represent the most commonly described methods in both the research literature and industry specifications.

## 2. Preservation Techniques

The tissue and cells that are preserved and stored must remain viable and exhibit biological activity after thawing. Challenges associated with preservation encompass the freezing method, cryoprotective agents, cell concentration, and specific types of cells subjected to freezing. Biobanks must also assess whether it is more effective to isolate and expand the cells before freezing or to freeze the tissue and subsequently isolate the cells after storage. Suboptimal cryopreservation may result in cell variation, diminished cellular functionality, decreased cell yield, and potential genetic or epigenetic alterations from the original cell line [11].

### 2.1. Cryopreservation

Cryopreservation is the most commonly employed method, wherein cells, tissues, or other biological constructs are preserved by being cooled to extremely low temperatures. This process initiates a phase change where liquid and aqueous materials freeze to form ice. Cells can undergo slow freezing using a rate-controlled freezer or rapid freezing through non-controlled rate methods. The rate-controlled freezing process involves slowly freezing cellular material at a rate of 1–2 °C/min down to approximately −40 °C. Subsequently, the freezing pace is accelerated, reaching about 3–5 °C/min, until a temperature of −120 °C is attained [12]. This extended process necessitates safety monitoring. Once at the desired temperature, the samples are stored either at −80 °C using solid carbon dioxide/dry ice or at −196 °C using liquid nitrogen [13]. Additionally, commercially available cryobags, cryoboxes, and cryotubes assist in regulating the freezing temperature of the enclosed product, emulating the effects of a rate-controlled freezer [14]. Moreover, there are uncontrolled freezing methods wherein the material is directly stored in a −80 °C freezer without careful control of the freezing temperature [15].

### 2.2. Vitrification

Vitrification is an ultra-rapid cryopreservation method wherein the entire biologic cell solution solidifies without the formation of ice crystals [16]. This process involves swiftly cooling the biologic material from 37 °C to −196 °C in less than 1 s. Vitrification finds widespread use in reproductive clinics, particularly for vitrifying embryos and blastocysts for storage [17]. The technique demands elevated concentrations of cryoprotectants compared to slow freezing and necessitates specialized equipment. However, vitrification is not commonly employed for preserving biologic products, except in the context of extracellular vesicles.

### 2.3. Lyophilization

Lyophilization, or freeze drying, involves freezing the cellular material, reducing the pressure, and applying heat to sublimate the frozen water. While lyophilization is extensively utilized in pharmaceutical applications for preserving proteins and liposomes, it is less commonly employed with cells [18]. Biomolecules such as proteins, DNA constructs, and liposomes are effectively preserved through the lyophilization process; however, mammalian cells typically do not survive the drying phase [19].

### 2.4. Cryoprotective Agents

Cryoprotective agents, or freezing additives, are often introduced to biological samples before freezing to counteract cell dehydration, cell lysis, and crystal formation, all of which can impact cell viability [20,21]. These agents can be categorized into two groups: small-molecular-weight penetrating cryoprotective agents (CPAs) and high-molecular-weight non-penetrating agents [22]. Cryoprotection revolves around avoiding or minimizing intracellular freezing and reducing damage to the cells [23]. Penetrating cryoprotecting agents, such as dimethyl sulfoxide, glycerol, ethylene glycol, and propylene glycol, are small molecules capable of crossing the cell membrane [24]. These molecules cause water to osmotically evacuate the cells slowly, leading to cellular dehydration and preventing issues related to ice crystal formation. A similar osmotic transition occurs in reverse during the removal of cryoprotective agents, as water re-enters the cells faster than the agents exit [25]. Non-penetrating cryoprotectants, including large molecules like sucrose, trehalose, and polyvinylpyrrolidone, usually polymers or sugars, operate similarly to penetrating cryoprotectants. They draw water out through osmosis without entering cells [26]. Trehalose, a disaccharide and a non-penetrating cryoprotectant, is commonly used in cryopreservation. Non-penetrating cryoprotectants are typically less toxic than penetrating ones at the same concentration [27]. These CPAs must maintain osmotic balance to prevent cellular damage, as cells either shrink or swell beyond tolerated limits during freezing.

Dimethyl sulfoxide (DMSO), a penetrating cryoprotective agent (CPA), serves as the standard cryoprotectant added to the culture medium for preserving and storing biological material [28]. Concentrations of this polar aprotic solvent (i.e., a solvent that cannot donate a hydrogen bond) have shown favorable effects in preserving viable cells compared to other agents like glycerol [29] and polyethylene glycol [30]. DMSO’s high membrane permeability allows it to easily enter cells and facilitate water escape. However, higher concentrations of DMSO can be detrimental to cells, directly impacting cellular function and growth [31,32]. Clinically, a notable side effect of DMSO injection is a garlic-like odor and taste resulting from the pulmonary excretion of DMSO as dimethyl sulfide [33]. This can persist in the body for up to 2 days, causing malodor and discomfort [34]. DMSO induces histamine release, leading to flushing and allergic reactions, which can have potential adverse effects in humans at higher concentrations [35]. Research efforts have been undertaken to identify potential alternatives to DMSO as a CPA or to reduce the concentration of DMSO used [36].

## 3. Individual Cell Therapy Preservation

### 3.1. PRP

Platelet-rich plasma (PRP), which is derived from autologous whole blood, undergoes processing to concentrate platelets. PRP products do not contain stem cells, and the FDA mandates a minimum of 250,000 platelets per microliter for a substance to be classified as PRP [37]. Additionally, PRP comprises a diverse array of biomolecules, including cells, proteins, cytokines, and growth factors. The bioactivity of PRP is attributed to the elevated concentrations of released growth factors [38]. Administered as a point-of-service care plan, the PRP procedure involves drawing the patient’s whole blood, processing it, centrifuging it, and then reinjecting it as PRP during the same visit [39].

Cryopreserving PRP is a commonly utilized method for long-term storage. In this process, PRP samples are mixed with 6% DMSO and placed in an uncontrolled freezer for storage at −80 °C [40]. In vivo survival and functional studies were conducted using 51Cr radioactive labeling. Post-thaw transfused platelets exhibited 70% similarity to fresh platelets, with no significant differences in bleeding suppression between the two groups [40]. In a different study comparing short-term storage, specifically 28 days at either −30 °C or −80 °C, there were no significant differences except for severe damage to the morphology and function of mitochondria in the warmer environment [41]. Human platelets can remain viable in storage for two to three years after cryopreservation using a rate-controlled freezer and a CPA at 6% [42] and 5% [43] DMSO. After 2 years in storage, platelet recovery post-thawing was approximately 75%, and the platelets demonstrated biological activity by reducing bleeding in thrombocytopenic patients [42]. After 3 years in storage, the recovery rate was 46%, and the infused platelets still demonstrated biological activity post-transfusion [43]. Equine-derived PRP samples, cryopreserved with 6% DMSO, were compared to PRP cryopreserved with trehalose [43]. The samples were stored in liquid nitrogen at −196 °C for 14 days. Here, 6% DMSO showed a higher platelet recovery rate (58%); however, there was a significant loss of TGF-β1 in all samples except fresh PRP [44].

Freezing and freeze drying are the most commonly employed methods for preserving and storing PRP. Wolkers et al. [45] reported no damage to freeze-dried platelets when loaded with the sugar trehalose. Shiga et al. [46] observed that PRP left at room temperature quickly degraded, while platelets that were freeze-dried and frozen at −80 °C maintained constant platelet counts. Further testing revealed that only the freeze-dried PRP group still maintained baseline levels of growth factors after 8 weeks [46]. Queiroz da Silva et al. [47] demonstrated that lyophilized PRP had similar concentrations of growth factors after 7 days of storage compared to fresh PRP, and lyophilized PRP exhibited biological activity through the induction and proliferation of fibroblasts. Freezing and thawing PRP released slightly lower levels of growth factors compared to the fresh preparation, with longer freezing times inducing a more significant decrease in growth factor release [48]

It is noteworthy that there is no standardized protocol for PRP preparation or reporting procedures, as indicated in Table 1 [49,50]. The lyophilization process differed between these studies, involving various buffers and reagents [51]. One of these studies measured the effects of long-term storage, with the longest time tested after storage being 8 weeks [46].

### 3.2. Mesenchymal Stem Cells (MSCs)/Stromal Cells/Progenitor Cells

Mesenchymal stem/stromal cells (MSCs) are multipotent adult stem/stromal cells found in various tissues, being commonly harvested from bone marrow, adipose tissue, and placental products [52]. Their biological activity stems from immunoregulatory and anti-inflammatory properties, being exerted through paracrine and small-molecule signaling [53]. These cells are often drawn and injected autologously to minimize the possibility of immune rejection [54] and to comply with current regulations on cellular therapies set by the United States Food and Drug Administration (FDA) [55].

The definition of MSCs is subject to ongoing refinement. According to a recent position paper from the International Society of Cellular Therapy (ISCT), the acronym MSC is still endorsed, but the ISCT recommends more detailed criteria to identify the tissue-source origin of cells. Researchers are encouraged to refrain from using the term “stem cells” unless evidence of stemness is supported by both in vitro and in vivo data, along with functional assays demonstrating MSC properties [56].

## 4. Bone Marrow Stem/Stromal Cells (BMSCs)

The bone marrow aspirate contains growth factors and various blood cells, including hematopoietic stem cells (HSCs). Mesenchymal stem cells are present in low numbers, with only 0.01–0.001% of the harvested cells meeting ISCT MSC criteria [57]. HSC products, including bone marrow products, are typically preserved through slow freezing with a rate-controlled freezer and cryopreservation solutions such as DMSO, glycerol, plasma proteins, salt, sugar, and hydroxyethyl starch (HES) [58].

In a comparison study, an uncontrolled freezing method was found to be safe, yielding equally viable cells post-thaw with similar amounts of cell loss, including CD34+ loss. The only significant difference observed was lower numbers of colony-forming units compared to a controlled-freeze method [59]. In a longer-term study (1 year +) comparing the two freezing methods, no significant differences were found in platelet or neutrophil recovery. Both freezing methods were considered safe and allowed for sustained, long-term engraftment of hematopoietic stem cells without increasing the risks of transplantation [60,61].

The standard cryoprotectant used for the processing and storage of human bone marrow is DMSO (10%) in culture media [62]. Liu et al. [63] found that a reduced concentration of the DMSO freezing solution (comprising 7.5% DMSO, 2.5% polyethylene glycol (PEG), and 2% albumin) can be comparable to the commonly used 10% DMSO solution in terms of the cell viability, apoptotic percentage, metabolic activity, proliferation, and differentiation capacities of BMSCs. By reducing DMSO concentrations, Liu et al. observed a minimal loss in cell viability (from 82.9% of the control to 82.7%) after a 1-week freeze. However, another study demonstrated that having concentrations of the DMSO freezing solution at 5% and 10% resulted in more live and viable human BMSCs (72% and 80.2%, respectively) after 5 months of storage [64].

In a survey of 95 European Bone Marrow Transplantation centers, all 95 responders exclusively utilized DMSO as their cryoprotectant. Among these, 78 centers used a 10% DMSO concentration, 9 centers used 5% DMSO, and 2 centers used 7.5%, while the remaining centers employed various concentrations, including 20%, 8%, 7%, 6%, 5.5%, and 2.2% [65]. A majority of these responding centers (60%) reported encountering issues related to DMSO toxicity post-transplantation. A summary of these studies can be found in Table 2.

DMSO is deemed a class 3 solvent by the FDA, which is the safest category with low toxic potential at levels normally accepted in pharmaceuticals [66]. DMSO is typically not removed from the cryopreserved stem cell before infusion, which may lead to adverse effects such as skin flushing, gastrointestinal issues, or cardiopulmonary complaints [67]. Severe adverse events are rare but have been reported from patients. Symptoms are mainly neurological events like seizures, encephalopathy [68], strokes, and cerebral hypofusion [69]. Most of these patients were infused with cells stored in 10% DMSO. Reducing the amount of DMSO reduces the problem of side effects, although washing the cells did not completely ameliorate issues [70]. The exact pathophysiology of these adverse events is unclear, and there is a possibility that these cases may be idiosyncratic. Users of these cellular therapies should be aware of the possible issues with these cryoprotective agents.

## 5. Adipose Stem/Stromal Cells/Progenitor Cells (ASCs)

Human adipose tissue is easy to access, is abundant, and contains more MSCs per unit volume compared to bone marrow [71]. The tissue is then processed through either mechanical emulsification, mechanical separation, or enzymatic digestion to obtain a heterogeneous mixture of cells including adipocytes, stromal vascular fraction, MSCs, endothelial cells, pericytes, fibroblasts, and hematopoietic cells [72,73]. Adipose tissue is regularly discarded from liposuctions and other surgical procedures and could be a promising source of stem cells.

Studies on the cryopreservation and storage of human adipose-derived stem cells have shown favorable results, as shown in Table 3.

Using enzymatic digestion, Minonzio et al. [74] isolated the stromal vascular fraction (SVF) from adipose tissue and mixed it with a cryopreservation agent containing 5% human albumin solution with 5% DMSO before using a rate-controlled freezer to store the solution in liquid nitrogen. After thawing the cells, the adipose stem/stromal cells retained 85% of their cell viability with normal proliferative capacity compared to fresh controls and were able to grow and differentiate into mesenchymal-specific lineages [74]. In a study comparing different cryoprotectant agents for ASCs, trehalose, DMSO, and fetal bovine serum (FBS) were used at different concentrations to determine their effects [75]. After three months of storage in liquid nitrogen, all groups maintained their functionality post-thaw, preserving their immunophenotype, differentiation potential, and proliferation, as assessed in vitro. However, cells preserved in DMSO had a much higher cell viability compared to trehalose. These findings suggest that storage in 5% DMSO without FBS may be ideal due to the low cytotoxicity and higher rates of cell viability compared to standard cryomedium of 10% DMSO + 90% FBS [75]. In a 4 year-long study, enzymatically digested adipose was frozen in a DMEM/F12 solution with 10% DMSO and stored in liquid nitrogen. After thawing, the cells showed high viability (95%), were successfully propagated through eight passages without losing their fibroblast-like morphology, and still possessed significant immunosuppressive properties [76]. In one 10-year freezing study, adipose was enzymatically digested, suspended in a cryoprotectant medium containing 10% DMSO in FBS, and placed in a rate-controlled freezer before finally entering long-term storage in liquid nitrogen [77]. The samples were stored for a decade before being thawed and tested for surface marker staining, RNA isolation, and differentiation. The immunophenotype and viability of ASCs did not change significantly compared to fresh ASCs, but osteogenic differentiation and hematopoietic markers decreased with the freezing process [77].

## 6. Gestational Tissue

Human gestational tissue represents a valuable and promising source of MSCs. A mature placenta consists of both fetal and maternal parts. The fetal parts are more commonly used in the field of regenerative medicine and include the chorionic plate, amnion, umbilical cord, and Wharton’s jelly found within the umbilical cord [78]. The most common product that is harvested and stored from the placenta is cord blood (CB). This blood is found in the umbilical cord after birth and is a rich source of hematopoietic stem and progenitor cells [79]. The United States Food and Drug Administration (FDA) has approved the use of cord blood in “hematopoietic stem cell transplantation” procedures (i.e., for the treatment of blood cancers such as leukemia, lymphoma, and other disorders of the blood and immune system) [80]. Because of the high demand for cord blood and the lack of regular access, 400+ cord blood banks have been formed around the world, storing millions of units of cord blood [81].

Optimal processing, cryopreservation, and storage are critical to ensure the safety and viability of the CB cells. Typically, the cord blood cells are sedimented from the mononuclear cells and leukocyte-rich plasma and subsequently mixed with a cryopreserving agent, usually 10% DMSO. The CB cells are then frozen slowly in a rate-controlled freezer until they are finally stored in liquid nitrogen at −156 °C (vapor phase) or −196 °C (liquid phase) [82]. This method of freezing has been used for a long time because it maintains high cell viability and clonogenic potential even after 15 years in storage [83]. However, just like bone marrow, there is research being conducted to find alternative agents (i.e., sucrose, trehalose [84], and dextrose [85]) or reduced concentrations of DMSO for cryopreservation. These alternative cryopreservation agents are not widely used in clinical practice.

Cord blood banks have finite space, and volume reduction is a widely used procedure in CB banking to remove the plasma and red blood cells from the frozen sample. These processing techniques range from centrifuging the blood to concentrate the cells, using sedimentation agents such as hydroxyl-ethyl starch (hetastarch), or processing them through automated systems (i.e., Sepax (Biosafe, Switzerland) and AutoXpress (ThermoGenesis, Rancho Cordova, CA, USA)) [86]. From Nikiforow et al.’s [87] survey of 34 CB banks, all banks utilized rate-controlled freezing; however, 41% of banks stored CB in the liquid phase of nitrogen, 38% in the vapor phase, and 21% in either phase. The CB banks also reported a variety of processing techniques, with a majority using automated processing systems [87]. Despite cord blood banks existing for decades, there are still no standardized protocols for cryopreservation, processing methods, or storing conditions [87].

Cord and placental tissues have garnered significant interest, since the MSCs isolated from these tissues could be considered the perfect candidates for cellular therapies and regenerative medicine. These MSCs have a fetal origin and, as a result, have a higher proliferative potential and greater differentiation plasticity [88,89]. Placental tissue has a long history of use as a biological dressing to treat burns and wounds [90]. In addition to neonatal cells, the placental membrane also contains structural matrix and growth factors that lend their regenerative properties. In commercial use, most placental membrane products are cryopreserved and devitalized without live cell products [91]. Grafix^®^ (Osiris Therapeutics, Inc., Columbia, MD, USA) is a cryopreserved human placental membrane, and it is the only commercially available placental membrane product to contain viable endogenous cells [92]. The cryopreservation method involves freezing the tissue in a DMSO solution at a controlled cooling rate. After storage at −80 °C for up to 3 months, the product has been reported to have an >80% cell viability and high levels of anti-inflammatory activity post-thaw [93].

There are two main strategies to isolate placental MSCs from the tissue. The explant method [94] seeds tissue fragments on a dish to grow cells, while enzymatic digestion [95] uses collagenase, hyaluronidase, or trypsin to separate the cells from the connective tissues. The explant method has been shown to yield higher levels of growth factors and have a higher cellular yield [96]. In a comparison test for post-thaw viability, the cells from the explant method had a higher viability and recovery rate than enzymatically digested cells after recovery from a 6-month liquid nitrogen storage period [97]. This study could not recover any viable MSCs from directly cryopreserved tissue fragments of placental tissue [97]. In a long-term study of freezing cord tissue MSCs, after five years of liquid nitrogen storage, thawed cord tissue (CT) MSCs had a lower plating efficiency and longer doubling time; however, they were found to still be clinically useful after in vitro expansion. Freezing cord tissue directly did not yield favorable numbers, as only less than 20% of recovered cells were viable [98]. These studies indicate that improved recovery rates for gestational cells need to be further researched for future clinical applications.

## 7. Extracellular Vesicles/EVs

Extracellular vesicles (EVs) are composed of a lipid bilayer and contain a cargo of various biomolecules including proteins, nucleic acids (mRNA, DNA, microRNA, and non-coding RNA), lipids, and metabolites [99]. Exosomes are a type of EV that are defined by the International Society for Extracellular Vesicles to have endosomal markers (CD9, CD61, CD83, ALIX, and TSG) and a size characterization of 30–100 nm [100,101]. Exosomes are secreted by every cell type, and their cargo reflects the biological state of the parent cells [102]. They act as messengers and transfer their cargo content to other cells, acting in a paracrine or even an endocrine manner to modify the behavior of adjacent or distant cells [103].

Exosomes are not living cells but instead are membrane vesicles formed by inward budding. Common methods of exosome isolation and purification employ ultracentrifugation, ultrafiltration, size exclusion, precipitation, immune isolation techniques, and microfluidic techniques [104]. The ultracentrifugation approach is a common way of isolating exosomes but has numerous drawbacks, including being highly labor intensive, time consuming, requiring a large amount of starting material, and having low exosome yields [105]. EVs suffer from aggregation and flocculation issues when stored in physiological saline solutions and degrade during freezing/thawing, affecting their physical membrane and biomolecular cargo [106].

Compared to colder temperatures (−70 °C) or room temperature, storage at 4 °C showed the least amount of protein, RNA, and exosome marker degradation [107]. Cheng et al. [108] conducted experiments on measuring the effects of pH, temperature, and freezing–thawing on exosomes. A pH around 7, storage at 4 °C, and lower cycles of freezing and thawing were ideal for storage. Changes within these testing conditions resulted in the degradation of exosome-associated proteins and RNA, as well as a decrease in the cellular uptake of these exosomes [108]. Exosomes stored at these below-freezing temperatures displayed a shrinking in their morphology, indicating a possible issue with aggregation and cryodamage, leading to the degradation of the exosome [109]. Trehalose has shown effectiveness when added as a storage buffer (25 mM), leading to a reduction in EV loss during storage at both at 4 °C for one day or −80 °C for up to one year [106].

Lyophilization, or freeze drying, is a commonly used process in ensuring the long-term stability of liposomes and has been investigated as an alternative to simply freezing as a method of preserving exosomes [110]. Exosomes that were lyophilized using a vacuum overnight and stored at room temperature were compared to exosomes stored at −80 °C for 1 to 4 weeks [111]. Without the addition of 50 mM of trehalose, the cryopreserved exosomes formed aggregates, which led to damage to the cellular morphology. However, when comparing lyophilized exosomes with trehalose and cryopreserved exosomes with trehalose, there was no significant difference in post-thaw in vitro testing. Both conditions had similar profiles of protein, exosomal markers, and RNA, and both had little effect on the pharmacokinetics of exosome uptake after injecting into mice, indicating that lyophilization could be usable as a freezing alternative [111]. Frank et al. [112] compared the short-term storage conditions of −80 °C, 4 °C, room temperature, and freeze drying (lyophilization) with various cryoprotective agents. In the study, trehalose as a CPA and mannitol performed similarly, both improving EV stability and morphology. The addition of PEG led to the aggregation of particles and a decrease in the protein concentration. For the freezing techniques, lyophilization had the smallest increase in size (aggregation) and had the most preserved enzymatic activity after a month of storage [112]. These studies can be found in Table 4. Further studies are needed to elucidate the variables of preservation to create a gold standard for exosome storage.

## 8. Discussion

Cellular therapies are increasingly integrated into patient care, requiring proper storage and preservation technology to transition from development to clinical application. The manufacturing and delivery of these therapies rely on cryopreservation and frozen storage to ensure safety, efficacy, and product optimization before administration [113,114]. Suboptimal yield of post-thaw cellular therapies is common and accepted due to the cells’ ability to be further expanded despite the risk of reduced viability, diminished function post-transfusion, and potential batch-to-batch variability [115].

The rising prevalence of chronic orthopedic conditions necessitates various biologic injection treatments. Given the complexities of modern society, it is crucial for treating clinicians to understand the processing of the new biologic revolution in medicine, particularly in musculoskeletal medicine. Therefore, it is critical that treating physicians comprehend the sourcing, content, and processing of these evolving biologic cell treatments. A lack of standardization in the biologics industry; variations in processing techniques, freezing times, and freezing techniques; and the use of cryopreservation agents can result in significant functional differences between samples post-thaw. Various viability assays are employed to measure the biological cell activity, making technique comparisons challenging. Common tests include counting cell numbers, measuring proliferation rates, and conducting growth factor release assays. It has been noted that measuring cell viability alone may lead to an overestimation of the activity in some biological materials. Instead, analyzing cell viability and recovery over a 24 h period may be necessary for a more accurate representation, allowing apoptosis to progress [116]. Additionally, other cell characteristics, such as cell morphology, epigenetic changes, and immunomodulatory behavior, might undergo alterations due to processing and preservation, necessitating further investigation. According to the 21st International Society for Cellular Therapy (ISCT) meeting, the consensus perspective for functional assays includes using flow cytometry, quantitative RNA analysis of selected gene products, and testing for immunomodulatory functions [117].

Advancements in freezing and thawing techniques, the selection and concentration of cryoprotective agents (CPAs), and other aspects of biopreservation could lead to improved survival rates and functionality of cellular sources, as well as enhanced biological activity. Research in this field not only contributes to the success of future clinical trials but also supports the expanding tissue bank industry. More studies are also needed to examine the biological properties of various orthobiologics after storage. A deeper understanding of the chemistry and biology involved in freezing and thawing processes is crucial for future progress and for identifying the safest and most effective cryopreservation methods to preserve function. Ultimately, the primary goals of future cryopreservation efforts should be to develop techniques that maintain the structural integrity of cells or tissues with minimal damage, followed by the standardization and optimization of these methods for widespread, routine use.

Currently, the most commonly used CPA for cellular products is DMSO. However, several studies have shown that higher concentrations of this chemical lead to greater adverse reactions [118,119]. Additionally, changes in the cell metabolism, cytoskeleton structure, and membrane fluidity has been observed with the addition of DMSO [120]. Various studies have attempted to reduce the concentration of DMSO or replace it as a CPA in cellular therapies, but the outcomes have been mixed [60,61,64]. Toxicity studies and their effects on cell behavior are necessary for other CPAs to ensure there are no adverse events or reactions post-thaw and after transfusion.

The literature indicates significant interest in preserving and storing cellular therapies for future use. The success of retrieving viable cells from thawed cellular therapies is influenced by factors such as the composition of the cryoprotectant medium, the freezing method, and the protocol used for cell isolation. Many current protocols show that post-thaw, cells remain viable, retain their phenotype, and function similarly to freshly isolated cells. Based on this review, the highest success and viability rates post-thaw include using a rate-controlled freezer with 6% DMSO for preserving platelet-rich plasma (PRP) [40,42]. Cellular treatments have also shown optimal outcomes with a rate-controlled freezer and 5% or 10% DMSO as a CPA. References [59,64,76,77,83] have demonstrated the best results when lyophilized with 50 mM or 4% trehalose to prevent aggregation and stored at room temperature [111,112].

## 9. Conclusions

The rapidly expanding field of regenerative medicine, particularly in new cell biology, involves various preservation methods. It is crucial for clinicians to understand this emerging area. However, the existing nomenclature and specific cryopreservation techniques often show confusion and imprecision in the literature. There is a lack of standardization in freezing times and cryopreservation techniques across different biological products, leading to variability in cell viability, apoptosis rates, and even biological efficacy after transfusion. Moving forward, the establishment of standardized protocols and measures of biological activity is essential to address data variability and promote collaboration within the research community.

## Figures and Tables

**Table 1:** Summary of preserved PRP studies.

Freezing Method	CPA Used	Viability Assay	Results	Reference
Frozen with CPA in refrigerator maintained at −80 °C	6% DMSO	Platelet concentration and transfusion of fresh and frozen platelets to patients	Recovery of freeze-preserved, washed platelet concentrates was 70% compared to control; Successfully stored at −80 °C for at least 6 weeks	[40]
Frozen with CPA in a freezer bag in a refrigerator maintained at −80 °C	6% DMSO	In vitro recovery of platelets and cultures and radioactive labeling of transfused platelets	Recovery of in vitro freeze–thaw wash recovery values of 85% In vivo recovery values of 31% 1–2 h after transfusion; Measured platelet life span was about 8 days	[42]
Controlled-rate freezing of −1 °C/min to −80 °C vs. placing them in the vapor phase (−120 °C) of a liquid nitrogen freezer	5% DMSO	In vitro platelet count and platelet survivability after transfusion	Recovery of frozen platelets was 46% or 11,800/microliter for frozen platelets and 25,900 for fresh platelets; Successful using these methods for greater than 3 yr with satisfactory post-transfusion increments	[43]
PRP stored at 4 °C vs. cryopreserved PRP stored in a liquid nitrogen canister at −196 °C, both with and without cryoprotectants	6% DMSO and 300 mM of trehalose	Platelet count, MPV determination, and TGF-β1 quantification	Recovery rate of PRP cryopreserved with DMSO was 58.08%; DMSO and trehalose failed to protect platelets against storage injuries caused by the cooling and cryopreservation processes (decreased platelet count and decreased growth factors)	[44]
Incubated in trehalose and frozen at a rate between −5 and −2 °C/min and then lyophilized at −35 °C	35 mM trehalose	Platelet recovery, aggregation studies, and protein structure analysis	Recovery rate of rehydrated platelets with trehalose was 85%; Reported platelets demonstrated almost identical optical density, thermotropic response of membranes, and overall protein secondary structure compared with control platelets	[45]
Frozen and stored at −80 °C vs. freeze-dried and stored at room temperature	None	Platelet counts, growth factor content, and platelet activation rate	Recovery rate of freeze-dried PRP was 80%; Demonstrated freeze-dried preserved platelet counts and growth factor levels, while freezing only maintained the platelet counts (80%)	[46]
Lyophilized for 20 h then stored in a −20 °C freezer	Buffer of tris, glycine, and sucrose	Analysis of platelet concentration, levels, and activities of growth factors	Recovery rates after lyophilization was 54%; Concentration of growth factors and ability to induce proliferation in fibroblasts after 24 h was similar for both lyophilized and fresh PRP	[47]

**Table 2:** Summary of preserved BM-MSC and HSC studies.

Freezing Method	CPA Used	Viability Assay	Results	Reference
Comparison of freezing blood progenitor cells in a controlled-rate freezer vs. uncontrolled	10% DMSO	Assessed viability, nucleated cell loss, mononuclear cell loss, loss of CD34+ cells, and recovery of colony-forming units	Viability of controlled-rate group of 88.8% compared to 89.7% uncontrolled rate No significant differences between 2 groups except for a significantly higher amount of colony-forming units in the controlled-rate technique	[59]
3 groups: uncontrolled freeze, controlled freeze, and liquid nitrogen preservation of peripheral blood hematopoietic stem cells at −80 °C	6% DMSO and 6% hydroxyl ethyl starch	Measured neutrophil recovery after hematopoietic recoveries following transplantation with cells	Median time required to reach 0.5 × 10^9^/L neutrophils was 12 (1–43), 11 (7–28), and 11 (5–22) days in the groups uncontrolled, controlled, and liquid nitrogen, respectively Uncontrolled-rate freezing and cryopreservation with 5% DMSO combined with HES at − 80 °C supports hematopoietic reconstitution compared to other groups even after prolonged storage	[54]
Comparison of controlled- vs. uncontrolled-rate freezing at −80 °C for peripheral blood hematopoietic stem cells	10% DMSO and 2.5% albumin concentration	Cell counts, cell viability, CD34+, and CFU-GM assay	Median time required to reach 0.5 × 10^9^/L neutrophils was 10 (range, 8–14) days in controlled and 11 (range, 7–22) days in uncontrolled Uncontrolled-rate freezing is slightly but statistically inferior to controlled rate in terms of speed of engraftment despite no differences in other variables	[55]
Controlled-rate freezing of BM-MSCs with variable DMSO concentrations as CPA	7.5%, 5%, and 2.5% DMSO with PEG or trehalose + albumin	Post-thaw cell viability, early apoptotic behavior, cell metabolic activities, and growth dynamics	Viability of BM-MSCs cryopreserved in controls were 82.7% (10% (*v*/*v*) DMSO), 83.8% (10% DMSO + 10% (*v*/*v*) FBS), and 90.2% (10% DMSO + 90% FBS), respectively Non-cryopreserved BM-MSCs showed a higher proliferation rate in comparison with the cryopreserved cells	[63]
Controlled-rate freezing of BM-MSCs with variable DMSO concentrations as CPA	10%, 5%, and 2% DMSO	Cell recovery, viability, apoptosis, proliferation rate, and expression of MSC markers	Viability of cryopreserved MSCs after 1 month with 2% DMSO was 91.7%, and viability of MSCs cryopreserved in CryoStor solutions with 5% and 10% DMSO was above 95%. Percentage of viable cells after 5 months was 72% and 80%, respectively	[64]

**Table 3:** Summary of preserved adipose-derived stem/stromal cell studies.

Freezing Method	CPA Used	Viability Assay	Results	Reference
Stromal vascular fraction cells were frozen in a 25 mL cryobag using controlled-rate freezing and stored in liquid nitrogen	5% DMSO and 5% albumin	Viability of adipose-derived stromal cells, cellular survival, differentiation ability, and colony-forming unit colonies	Viability of cells showed 85%, 180,890 cell/g yield, plus normal proliferative capacity and differentiation potential compared with fresh controls up to 193 days	[74]
P2 human adipose stem cells were frozen in cryovials, kept at −80 °C overnight, and transferred to liquid nitrogen (−196 °C) for 3 months in different combinations of CPAs	(1) 0.25 M trehalose; (2) 5% DMSO; (3) 10% DMSO; (4) 5% DMSO + 20% fetal bovine serum (FBS); (5) 10% DMSO + 20% FBS; (6) 10% DMSO + 90% FBS	Measured cell viability rates, cell phenotype, and proliferation rates	Specific viability was not reported; however, 5% DMSO without FBS may be an ideal CPA for the long-term preservation of ASCs, maintaining the cell phenotype, and its functional properties, and it is less cytotoxic and leads to a high rate of cell viability; ASCs preserved in 0.25 M trehalose showed the lowest cell viability	[75]
After 2nd passage, ASCs were frozen in an uncontrolled-rate freezer and stored in liquid nitrogen	10% DMSO and 20% autologous serum	Surface marker expression, differentiation potential, and immunosuppressive effect in vitro	Specific viability was not reported; ASCs cultured in the medium supplemented with 5% AS were propagated through 8 passages without the loss of fibroblast-like morphology, MSC surface marker expression, differentiation, and immunomodulatory potential even after double freezing and more than 4 years of cryopreservation	[76]
Cultured ASCs were frozen in cryovials in a rate-controlled freezer, transferred to liquid nitrogen, and stored for 10 years	10% DMSO and FBS solution	Post-thaw viability, stromal surface markers, and qPCR analysis	Mean cell viability for the donors in the short-term group was 79%, whereas it was 78% in the long-term group; osteogenic differentiation potential was decreased in long-term cryopreserved group; post-thaw viability of ASCs also remained intact after decade-long freezing process in relation to fresh ASCs.	[77]

**Table 4:** Summary of preserved exosome/extracellular vesicle studies.

Freezing Method	CPA Used	Viability Assay	Results	Reference
Exosomes were aliquoted in cooled 1.5 mL microcentrifuge tubes and stored at room temperature, 4 °C, and −70 °C for 10 days	None	Exosomal protein RNA and exosome markers were analyzed	Protein and RNA amounts were most reduced at room temperature, there was major loss of CD63, and exosomes were clumped together at −70 °C	[107]
Exosomes were stored at different conditions, including temperatures, pH levels, and levels of freezing	None	Measured cell viability rates, cell phenotype, and proliferation rates	Levels of exosomal proteins decreased in all groups, but degradation rate at −80 °C was the lowest; however, there was decrease in the exosome concentration at all temperatures and freeze thawing affected the exosomal membrane	[108]
Exosomes were stored for 4 days at +4 °C or −80 °C	None	Exosomal structure was assessed via protein content, dynamic light scattering, transmission electron microscopy, and charge density	Storage destabilizes the surface characteristics, morphological features, and protein content of exosomes; caused increases in diameter, significant reduction in zeta potential, and significant reduction in identifiable proteins	[109]
Comparison between exosomes stored at −80 °C and ones lyophilized and stored at room temperature	50 mM of trehalose	Analyzed protein and RNA contents and physicochemical and pharmacokinetic properties	Addition of trehalose reduced exosome aggregation and protected protein and RNA content post-thaw; lyophilization had little effect on pharmacokinetics after intravenous injection	[111]
Comparison of EVs in various storage conditions, including −80 °C, 4 °C, room temperature, and freeze drying (lyophilization)	4% trehalose	Analyzed stability and size of EVs, functionality, and protein and RNA contents	No immediate effect on size during freeze drying compared to storage at −80 °C; there was a decrease in the activity at 4 °C and −80 °C, which was less pronounced for EV samples lyophilized with 4% trehalose	[112]
